# Diagnostic uncertainty in primary care: what is known about its communication, and what are the associated ethical issues?

**DOI:** 10.1093/fampra/cmab023

**Published:** 2021-04-28

**Authors:** Caitríona L Cox, Benjamin M Miller, Isla Kuhn, Zoë Fritz

**Affiliations:** 1 THIS Institute (The Healthcare Improvement Studies Institute), Department of Public Health and Primary Care, University of Cambridge, Cambridge, UK; 2 St Mary’s Surgery, Ely, UK

**Keywords:** Communication, education, ethics, patient-centred care, patient–doctor relationship, uncertainty

## Abstract

**Background:**

Diagnostic uncertainty (DU) in primary care is ubiquitous, yet no review has specifically examined its communication, or the associated ethical issues.

**Objectives:**

To identify what is known about the communication of DU in primary care and the associated ethical issues.

**Methods:**

Systematic review, critical interpretive synthesis and ethical analysis of primary research published worldwide. Medline, Embase, Web of Science and SCOPUS were searched for papers from 1988 to 2020 relating to primary care AND diagnostic uncertainty AND [ethics OR behaviours OR communication]. Critical interpretive synthesis and ethical analysis were applied to data extracted.

**Results:**

Sixteen papers met inclusion criteria. Although DU is inherent in primary care, its communication is often limited. Evidence on the effects of communicating DU to patients is mixed; research on patient perspectives of DU is lacking. The empirical literature is significantly limited by inconsistencies in how DU is defined and measured. No primary ethical analysis was identified; secondary analysis of the included papers identified ethical issues relating to maintaining patient autonomy in the face of clinical uncertainty, a gap in considering the direct effects of (not) communicating DU on patients, and considerations regarding over-investigation and justice.

**Conclusions:**

This review highlights significant gaps in the literature: there is a need for explicit ethical and patient-centred empirical analyses on the effects of communicating DU, and research directly examining patient preferences for this communication. Consensus on how DU should be defined, and greater research into tools for its measurement, would help to strengthen the empirical evidence base.

**Key Messages**

- Diagnostic uncertainty is common in primary care.
- Communication about diagnostic uncertainty to patients is often limited.
- This review highlights significant gaps in the literature.
- There is variation in how diagnostic uncertainty is defined and measured.
- More ethical and patient-centred empirical analyses are needed.

## Introduction

Primary care sees undifferentiated presentations, with disorder arising from biological, psychological and social domains and any combination thereof (1). Medical education often emphasizes certainty, creating the impression that there is always a ‘right answer’; this is not the reality in clinical practice (2,3). Indeed, primary care clinicians have reported that 100% certainty of a diagnosis in general practice does not exist (4); they experience uncertainty more than any other speciality other than psychiatrists (5).

In primary care, patients present with undifferentiated symptoms: the focus must be on exploring the full range of diagnostic possibilities, as opposed to merely considering one specific diagnosis. The extent to which primary care clinicians communicate this *initial* diagnostic uncertainty with their patients has received relatively little attention (in contrast to communication surrounding medically unexplained symptoms, which typically occurs after more extensive investigation and has been examined more widely) (6,7). An increasing emphasis on shared decision-making in primary care (8–10) in response to uncertain prognostic or treatment outcomes may have an impact on expectations—from both patients and clinicians—about information-sharing in the diagnostic process. Indeed, new guidance from the GMC recommended that if doctors are uncertain about the diagnosis, they should explain this to the patient (11).

A recent literature review of the management of diagnostic uncertainty in primary care was performed by Alam *et al.*; their main focus was ‘*to explore which types of skills or strategies physicians use to manage diagnostic uncertainty*’ (12). They concluded that in the extant literature, primary care clinicians manage diagnostic uncertainty in a variety of ways. These can be broadly categorized as cognitive, emotional and ethical. The ethical aspects identified were primarily concerned with the communication of diagnostic uncertainty; their search was limited to how physicians approached this challenge.

This review builds on this work by focussing on the *communication* of diagnostic uncertainty in primary care: how and why it is (not) communicated to patients, and what are the effects of this (non) communication?

We therefore reviewed the literature to answer the following research questions:

(1) What is known about the communication of diagnostic uncertainty in primary care?
(2) What are the ethical issues associated with communicating (or not communicating) diagnostic uncertainty in this setting?

## Methods

A systematic review framework was employed to ensure a robust and replicable searching strategy and clarity of data extraction. Critical interpretive synthesis (13) and further ethical analysis were used to analyse a complex body of literature that combined both qualitative and quantitative studies spanning multiple disciplines.

### Protocol

The review was registered on the PROSPERO database (registration ID CRD42018099561). PRISMA guidelines were followed (see Supplementary Materials).

### Identification of studies

Through trial searches, broad search criteria were utilized to capture all relevant literature that referenced: primary care AND diagnostic uncertainty AND [ethics OR behaviours OR communication]. The full search strategy can be found in Supplementary Materials.

Studies were identified by searching electronic databases (Medline via OVID, Embase via OVID, Web of Science and Scopus). Searches initially covered papers from 1988 to 2018, and were rerun to extend to 23.09.2020. Reference lists of included studies were reviewed for additional papers. A complete record of all identified articles was kept on a managed reference database (EndNote X8).

### Inclusion criteria

All primary research study designs in English were considered which addressed (either explicitly or implicitly) diagnostic uncertainty and either communication or ethics, which were set in, or primarily concerned with, primary care.

### Exclusion criteria

Abstracts/conference proceedings were excluded. Studies were excluded which were set wholly outside of primary care, or which focussed on: a specific single disease; resuscitation decisions or acute care in disaster situations; communication with non-qualified physicians; uncertainty in other contexts, (e.g. therapeutic, scientific or prognostic uncertainty); medically unexplained symptoms; communication between health care professions; communicating surrounding screening programmes.

### Study selection

Titles and relevant abstracts were screened for eligibility criteria for inclusion (BM 1988–2018; CLC 2018–2020 search); ZF co-screened 10% of all papers to ensure consistency. BM or CLC and ZF screened all those included for full text analysis and reference lists. Disagreements were resolved through face-to-face discussions.

### Data extraction and risk of bias

A standardized data extraction form was developed and piloted (Supplementary Materials). Data included: country of origin and the setting; methodologies used; limitations and biases; key results. Data were cross-checked by the three reviewers and any disagreements were resolved by discussion.

### Planned methods of analysis

The heterogeneous nature of the research and literature identified by the searches did not lend itself to meta-analysis. A critical interpretive synthesis (13) and ethical analysis were thus performed in addition to the systematic review in order to identify unifying or explanatory themes, and ethical issues emerging from the literature. A full account of both the merits of and processes involved in critical interpretive synthesis can be found here (13,14); briefly, it involved an analysis and familiarization with the studies, discussion of the extracted data and consideration of the themes that arose. Critical examination of the ways the literature had constructed the nature of diagnostic uncertainty and its management helped iterate the research questions, leading us to focus more tightly on communication. Inclusion criteria permitted the inclusion of qualitative, quantitative and mixed research including methodologically weaker studies that were of clear relevance. We utilized an inductive approach to identify recurring themes and develop a synthesizing argument. The ethical analysis was conducted first using the ‘four principles’ (beneficence, non-maleficence, autonomy and justice) (15) as a guide; an iterative process was utilized in which emerging ethical issues were identified and then discussed, with attention to cross-cutting and repeating themes.

## Results

**Figure 1.**
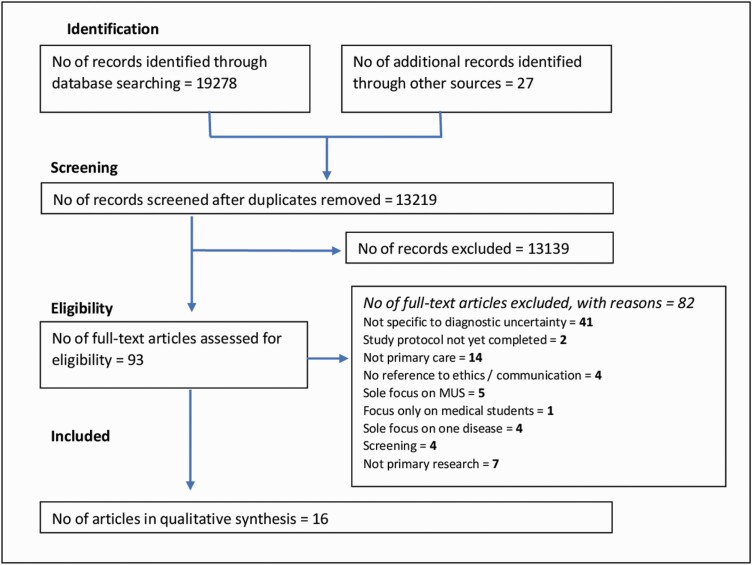
PRISMA flow diagram.

A summary of the study screening and selection process can be seen in Figure 1; characteristics of the included literature can be seen below, followed by an exploration of each research question.

### Characteristics of included literature

Table 1 shows details of articles included in the qualitative synthesis, their methodologies and their limitations.

**Table 1.** Details of included papers identified during search 1988–2020

Authors and title	Aims/objectives	Country of study	Article type, research methods, data collection analysis and limitations	Key results
*Qualitative research*				
Physician responses to ambiguous patient symptoms. Seaburn *et al.* (22)	To examine how primary care physicians respond to ambiguity.	USA	Type: primary research—*qualitative* Methods: observational study of *n* = 23 GPs using standardized patients. Analysis: thematic analysis. Limitations: a relatively small sample of clinicians, utilizing qualitative analysis. Many not be generalizable to wider population of GPs. Use of standardized, as opposed to real, patients.	• Physicians’ responses to ambiguous symptoms were categorized into two primary patterns: high partnering (HP) (43%) and usual care (UC) (57%).
				• HP was characterized by greater responsiveness to patients’ expression of concern, greater acknowledgment of symptom ambiguity and solicitation of patients’ perspectives on their problems.
				• UC was characterized by denial of ambiguity and less inclusion of patients’ perspectives on their symptoms.
				• When confronted with ambiguous symptoms, study physicians almost uniformly either denied ambiguity or coupled acknowledgment of ambiguity with a directive.
Patients’ experiences of cancer diagnosis as a result of an emergency presentation: a qualitative study. Black *et al.* (26)	To capture in detail the experiences of patients whose cancer was diagnosed following an ED visit to understand how emergency presentations arise and identify where there is scope to improve outcomes.	UK	Type: primary research—*qualitative* Methods: interviews with *n* = 27 patients to assess their experiences of health care services before diagnosis. Analysis: thematic analysis of interviews. Limitations: a limited number of potential participants were approached for interview. Results may not be generalizable to non-cancer patients.	• Patient concerns about discovering they have cancer, and about the harms or embarrassment of investigations, may have acted as particular incentives to adhering strongly to a benign diagnosis once given.
Challenges and strategies for general practitioners diagnosing serious infections in older adults. McKelvie *et al.* (25)	To explore issues GP face in the diagnosis of serious infection in adults.	UK	Type: primary research—*qualitative* Methods: semi-structured interviews *n* = 28 GPs. Analysis: framework analysis of interviews. Limitations: self-selection bias in recruitment, as well as hindsight bias in GP accounts of cases. The study is specific to older patients, therefore may not be generalizable to general population.	• Three main themes emerged: challenges leading to diagnostic uncertainty (patient complexity, atypical presentation, lack of knowledge about the patient); approaches to the patient (history-taking, physical examination and use of scoring systems), and management of diagnostic uncertainty (ordering investigations, and safety-netting).
				• Investigations were sometimes used to resolve diagnostic uncertainty, but availability and speed of result limited their practical use.
				• Clear safety-net plans shared with patients and their families helped GPs manage ongoing uncertainty—GPs both discussed safety-netting plans with a third party if required, and gave specific conditions when they would expect the patient to return.
				• Shared responsibility by safety-netting gave GPs less anxiety.
‘Chasing a Ghost’: factors that influence primary care physicians to follow up on incidental imaging findings. Zafar *et al.* (27)	To explore provider and patient characteristics that influence how primary care providers communicate and manage incidental image findings.	USA	Type: primary research—*qualitative* Methods: semi-structured interviews *n* = 30 primary care providers. Analysis: framework analysis of interviews. Limitations: this study was conducted at a single academic medical centre, which may limit the generalizability of these results. There was no patient involvement, so only able to give information on physician perspectives on decision-making.	• In order to eliminate uncertainty, some primary care providers felt compelled but frustrated to pursue costly follow-up for incidental imaging findings of limited clinical importance. Others did not act on findings that were unfamiliar or occurred in an unusual clinical context when follow-up recommendations were not given.
				• Some reported using a uniform approach to communicate and manage incidental findings, while others used pre-test probability to inform them on the significance of the finding, or adapted their approach according to their patient.
				• There was evidence of variation in how much information about the possible diagnosis causing incidental imaging findings primary care providers disclose to different patients.
“I got my diagnosis on a yellow post-it note”: young adult cancer patients’ experiences of the process of being diagnosed with cancer. Hauken *et al.* (29)	To explore how young cancer survivors experience the process of being diagnosed with cancer.	Norway	Type: primary research—*qualitative* Methods: a qualitative method founded on a phenomenological-hermeneutical approach was used, and included in-depth interviews with 20 young adult survivors (aged 24–35 years) with different cancer diagnoses. Analysis: thematic analysis of interview data. Limitations: small sample size. Findings cannot be generalized to the broader population. The study also had an underrepresentation of men and of the youngest age group (18–23 years).	• The participants’ experiences of the diagnosis process were elaborated according to three main themes: (i) ‘I felt something was wrong, but…’ (ii) ‘The traumatic uncertainty’ and (iii) ‘The day my world collapsed’.
				• ‘The traumatic uncertainty’ was further divided into two subthemes: (i) ‘But no one would tell’ and (ii) ‘To live in suspense’.
				• A common feature of this period was that the participants experienced a dearth of information and considerable worry and uncertainty; many did not fully understand the reasons for invasive and sometimes painful investigations.
				• Several participants had long periods awaiting investigation results, characterized by considerable anxiety.
Quality improvements of safety-netting guidelines for cancer in UK primary care: insights from a qualitative interview study of GPs. Tompson *et al.* (18)	To seek the insight of frontline GPs regarding proposed safety-netting guidelines for suspected cancer in UK primary care.	UK	Type: primary research—*qualitative* Methods: semi-structured interviews with GPs (*n* = 25). Interviewees were asked about their views and experiences of safety-netting and then presented with the safety-netting recommendations. Analysis: thematic analysis of interview data. Limitations: small sample of GPs, all from one region. 1/3 of the interviewees were personal contacts of the research team.	• GPs were supportive of initiatives to optimize safety-netting.
				• Sharing diagnostic uncertainty was thought to be helpful, but was only partially implemented.
				• GPs agreed that they should explain the uncertainty of the working diagnosis to patients, but suggested that their ability to do so was limited both by the length of consultations and by some patients preferring ‘black and white answers’.
				• Neither informing patients of all (including negative) test results nor ensuring recurrent unexplained symptoms are always flagged and referred were considered feasible.
Communicating cancer risk in the primary care consultation when using a cancer risk assessment tool. Akanuwe *et al.* (30)	To explore the perspectives of service users and primary care practitioners on communicating cancer risk information to patients, when using QCancer, a cancer risk assessment tool.	UK	Type: primary research—*qualitative* Methods: semi-structured interviews and focus groups with 36 participants (19 service users) and 17 primary care practitioners. Of the 19 service users, two had a previous diagnosis of cancer, and the rest had relatives or friends who had a previous diagnosis of cancer. Analysis: framework method of thematic analysis. Limitations: relative lack of diversity in the sample: all 19 service user participants were of White British ethnicity. Users all had personal experience of cancer, and their views might systematically differ from those without experience of a cancer diagnosis.	• This paper discusses the communication of uncertainty regarding potential cancer diagnosis, in the context of patients presenting with undifferentiated symptoms.
				• Participants suggested ways to improve communication of cancer risk information: personalizing risk information; involving patients in use of the tool; sharing risk information openly; and providing sufficient time.
				• A key theme was being open and honest: participants emphasized the importance of telling patients the truth about their health information, even if this led to increased worry.
				• Not being honest about the risk of cancer could affect trust between patients and practitioners.
Improving the quality of care and patient experience of care during the diagnosis of lupus: a qualitative study of primary care. Amsden *et al.* (28)	To better understand diagnostic delay and doctor–patient communication during the diagnosis of systemic lupus erythematous in patients without malar rash.	USA	Type: primary research—*qualitative* Methods: interviews with *n* = 8 primary care physicians. Analysis: thematic analysis. Limitations: small number of participants, self-selection bias, hindsight bias in recounting cases.	• There were five domains related to diagnosis: initial assessment and tests, initial diagnosis and empiric treatment, timeliness of diagnosis, communicating with the patient and opportunities for improvement.
				• In general, the physicians stressed the importance of being honest with the patient. Some would explain the approach to the diagnosis and outline the differential diagnosis.
				• The paper discusses the need to clearly communicate when a diagnosis is not yet final and when an ordered treatment is empiric in order to reduce diagnostic errors and delays.
				• Doctor–patient communication is critical to help the physician make sense of the symptoms, maintain trust and assure the patient that he or she is receiving appropriate care.
Acute low back pain management in general practice: uncertainty and conflicting certainties. Darlow *et al.* (24)	To explore GP beliefs about acute low back pain, and how these influence their clinical management.	NZ	Type: primary research—*qualitative* Methods: semi-structured interviews with *n* = 11 GPs. Analysis: thematic analysis. Limitations: small number of participants, centred around one presenting complaint.	•Specific diagnosis was often not seen as achievable or helpful in the diagnosis of low back pain.
				•GPs perceived patients as intolerant of uncertainty and wanting a diagnosis.
				•GPs either (i) gave a specific diagnosis in order to reassure patients, or (ii) were more open about the uncertainty.
				•Some participants provided diagnostic labels despite an inability to accurately diagnose, and against guideline recommendations. They felt simple musculoskeletal diagnoses were helpful to reassure (themselves as much as their patients) that pathology was not present, and to encourage exercise, which was therapeutic.
*Quantitative research*				
Doctors expressions of uncertainty and patient confidence. Ogden *et al.* (16)	To examine the impact of the way in which uncertainty was expressed (behaviourally versus verbally) on doctor’s and patient’s beliefs about patient confidence. Also examined the role of the patient’s personal characteristics and knowledge of their doctor as a means to address the broader context.	UK	Type: primary research—*quantitative* Methods: matched questionnaires sent to GPs and patients. Fourteen expressions of uncertainty were described, conceptualized as either behavioural (*n* = 8) or verbal (*n* = 6). Participants were asked about what effect they thought the expressions of uncertainty would have on patient confidence; they were asked to rate each of the expressions of uncertainty on a 5-point Likert scale ranging from `not at all confident’ (1) to `totally confident’ (5). Patients also asked how often they had been to see the GP in the past year, and how well they felt that they knew their GP rated on a 5-point Likert scale. Analysis: statistical analysis on GPs’ and patients’ ratings of the expressions of uncertainty. The data were also analysed using multiple regression analysis to explore the role of the patient’s personal characteristics and experience of their doctor in predicting their reaction to verbal and behavioural expressions of uncertainty. Limitations: use of analogue patients. Survey data asking participants to speculate on effects, rather than collecting actual effects of communicating uncertainty in real interactions.	• Verbal expressions of uncertainty such as ‘Let’s see what happens’ were viewed as the most potentially damaging to patient confidence, and both GPs and patients believed that asking a nurse for advice would have a detrimental effect.
				• In contrast, behaviours such as using a book or computer were seen as benign or even beneficial.
				• GPs and patients agreed about behavioural expressions of uncertainty, but the patients rated the verbal expressions as more detrimental to their confidence than anticipated by the doctors.
				• Patients who indicated that expressions of uncertainty would have the most detrimental impact upon their confidence were younger, lower class and had known their GP for less time.
*Mixed methods research*				
Communicating and dealing with uncertainty in general practice: the association with neuroticism. Schneider *et al.* (17)	To estimate the association with personality traits on handling of uncertain situations in general practice.	Germany	Type: primary research—*mixed methods* Methods: mixed methods approach: Qualitative analysis of focus group discussion. Interviews using cognitive think aloud technique. (10 GPs). Cross-sectional survey of GPs (228 responses). Analysis: statistical analysis (mean, standard deviation, ANOVA, Pearson correlation) of data set. Limitations: questionnaires developed with GPs who attend conferences and teach—bias towards those who these interests/critical thinking.	• Neuroticism was positively associated with all PRU scales ‘anxiety due to uncertainty’, ‘concerns about bad outcomes’, ‘reluctance to disclose uncertainty to patients’ and ‘reluctance to disclose mistakes to physicians’.
				• Neuroticism was negatively associated with the CoDU scale ‘communicating uncertainty’.
				• ‘Extraversion’, ‘agreeableness’, ‘conscientiousness’ and ‘openness to experience’ were significantly positively associated with ‘communicating uncertainty’.
Strategies for managing uncertainty and complexity. Hewson *et al.* (20)	To identify strategies involved in diagnosis and treatment of primary care problems that are uncertain and complex.	USA	Type: primary research—*mixed methods* Methods: (1)Observed videos (*n* = 10) of primary care physicians interacting with four standardized patients with complex/uncertain primary care problems, performed qualitative analysis to identify ‘strategies’. These strategies then rated by physicians in GIM (*n* = 19) on a 1–10 Likert scale for perceived importance in their primary care practice. (2)Transcripts of audiotapes of physician-standardized patient encounters analysed to record incidence of the strategies. Limitations: small numbers, identification of strategies for dealing with complexity (as well as uncertainty).	• Nine strategies were identified, and each was rated as important to primary care clinical practice.
				• Strategies include: eliminates alternative diagnoses by dealing with patient fears, giving reasons in the context of the patient’s belief system; and keeps diagnostic options open by making provisional diagnoses while keeping alternatives in mind.
‘Could this be something serious?’ Reassurance, uncertainty, and empathy in response to patients’ expressions of worry. Epstein *et al.* (21)	To describe physicians’ responses to patients’ worries, how their responses varied according to clinical context, and associations between their responses and patients’ ratings of interpersonal aspects of care.	USA	Type: primary research—*mixed methods* Methods: multimethod study. For each physician covertly audio recorded two unannounced standardized patient (SP) visits. SPs expressed worry about ‘something serious’ in two scenarios: straightforward gastroesophageal reflux or poorly characterized chest pain with MUS. Also patient surveys (*n* = 50) given to real patients in the waiting room for each physician, measuring interpersonal aspects of care (trust, physician knowledge of the patient, satisfaction and patient activation). Analysis: qualitative coding of these transcripts, followed by descriptive, multivariate and lag-sequential analyses. Limitations: use of SPs, only two clinical scenarios. Expressions of uncertainty ‘I don’t know’, as opposed to acknowledgement of diagnostic uncertainty.	• Biomedical inquiry and explanations, action, non-specific acknowledgment and reassurance were common, whereas empathy, expressions of uncertainty and exploration of psychosocial factors and emotions were uncommon.
				• Expressions of uncertainty (e.g. ‘I don’t know’) were the least frequently observed response.
				• Physicians’ expressions of uncertainty (‘I don’t know…’) were not associated with lower patient ratings of the physician.
Dealing with uncertainty in general practice: an essential skill for the general practitioner. O’Riordan *et al.* (2)	To discuss the importance of managing uncertainty in primary care and to propose educational interventions which can improve this.	European	Type: primary research—*mixed methods* Methods: mixed methods: literature review and expert focus group consensus. Limitations: details of methodology for focus group consensus not included in manuscript.	• Describes the management of uncertainty as an essential skill which should be included in educational programmes for both trainee and established GPs.
				• The literature on dealing with uncertainty focuses largely on identifying relevant evidence and decision-making.
				• Highlights that there is a need for GPs to accept some uncertainty in complex situations to avoid burnout.
				• Suggests that a good doctor–patient relationship is vital, creating trust and mutual respect, developed over time with good communication skills.
				• Recommends sharing the degree of uncertainty with the patient. Evidence-based medicine should be used, including discussion of probabilities where available.
				• Notes that SDM encourages sharing uncertainty with patients, but the effect of doing this on patients has not been thoroughly researched.
Identifying transparency in physician communication Robins *et al.* (19)	To categorize physician communication, to gain understanding of what patients want to know and skill in conveying that information.	USA	Type: primary research—*mixed methods* Methods: analysis and coding of 263 audiotaped interactions between 33 primary care physicians and their patients in eight community-based, primary care clinics. Analysis: qualitative examination of audiotapes, with some quantitative analysis (e.g. the proportion of time spent on different behaviours in the consultation). Limitations: wide variation in transparency utterances across physicians. Study was not able to determine whether study patients noticed or valued the types of transparency utterances identified or what these utterances might have contributed to patients’ perceptions of their physicians.	• Physicians proactively used five types of process transparency to preview speech and actions. Four types of content transparency were used to explicate diagnosis and treatment, demystify medical language and concepts and interpret biomedical information. Physicians spent the greatest proportion of clinic time explicating medical content.
				• The typology ‘diagnosis rationale’ (Sharing personal thinking about patient’s diagnosis, especially what symptoms or signs are suggestive of a condition or distinguish one possible condition from another) was one of those that physicians engaged least with as a proportion of clinic time (<1.0% mean encounter time).
The wrong diagnosis: Identifying causes of potentially adverse events in general practice using incident monitoring. Bhasale *et al.* (23)	To identify how diagnostic incidents occur and to understand their preventable causes.	Australia	Type: primary research—*mixed methods* Methods: GPs anonymously reported incidents of potential harm using free text or structured responses. In this paper, *n* = 142 diagnostic incidents were examined. Analysis: descriptive statistics of quantitative data, and thematic analysis of qualitative data. Limitations: hindsight bias, as results rely on the accuracy of GPs reporting incidents.	• Diagnostic incidents are often due to errors in judgement in the formation of a diagnosis Type A errors (inappropriately rejecting the correct diagnosis) and Type B errors (inappropriate hypothesis formation). Poor communication (both between health care professionals and between doctors and patients) often frequently contributes.
				• Ineffective communication to the patient about the need for further assessment/poor understanding about the diagnostic process was the main factor in five incidents.
				• Patients did not always know what was expected of them, e.g. a case of delayed appendicitis diagnosis, in which the patient was not adequately informed about the provisional nature of the initial working diagnosis of gastroenteritis.
				• In another case, a patient did not return for planned X-rays, having not understood risk of unhealed scaphoid fracture.
				• Improved communication between patient and doctor (by being clear about the diagnostic uncertainty) could reduce diagnostic incidents.

Sixteen publications were identified: nine qualitative studies, six mixed methods and one quantitative study. There was a western bias to the literature: six studies originated from North America, eight from Europe and two from Australasia.

(1) What is known about the communication of diagnostic uncertainty in primary care?
The lack of clarity over definition and measurement limits our understanding.

The literature identified in this review was heterogenous, both in conceptualization and measurement of diagnostic uncertainty.

A striking feature of the identified papers was a lack of clarity over ‘diagnostic uncertainty’ as a separate entity to other types of uncertainty which exist in medical practice (such as uncertainty in prognosis, or in treatment). None of the identified papers provided a clear definition of ‘diagnostic uncertainty’. Ogden acknowledged this, suggesting that the variation in how uncertainty is defined and operationalized could account for inconsistencies in the literature (16).

The variation in interpretation of diagnostic uncertainty has implications for the strength of the conclusions which can be drawn from the data: as studies conceptualized and measured diagnostic uncertainty differently, the extent to which findings can be compared or generalized is limited. For example, the communication of diagnostic uncertainty could be examined through measuring patient responses to their doctor admitting to a lack of knowledge: Ogden examined verbal expressions of uncertainty (e.g. ‘I don’t know’ and ‘I haven’t come across this before’), and behavioural expressions of uncertainty (e.g. checking information in a book/computer or asking a colleague for advice) (16). In other studies, communicating diagnostic uncertainty was considered to involve explicitly discussing with patients any uncertainty over the working diagnosis, and sharing thinking about the diagnostic process such as the reasons for investigations and the exclusion of differential diagnoses (17–19).

Other studies elided diagnostic uncertainty with ambiguity or complexity: Hewson used simulated patients, where the cases were ‘uncertain and complex, involving undifferentiated or incomplete clinical data, with both benign and life-threatening possibilities’ (20). Both Seaburn and Epstein similarly used simulated patients with multiple unexplained symptoms, designed to create diagnostic ambiguity (21,22).

In summary, although many of the papers identified in this review reported aspects of diagnostic uncertainty, the lack of consensus on definition or measurement of the construct makes meaningful analysis challenging.

1. Strategies for managing diagnostic uncertainty, including communication techniques for sharing uncertainty with the patient, are acknowledged as vital in primary care.

Many of the papers identified noted that diagnostic uncertainty is inherent in primary care (2,17,18,20,22,23). A number highlighted the importance of primary care clinicians developing skills for its management, including techniques for communicating uncertainty with patients (2,20,24,25).

Hewson identified nine techniques which primary care clinicians use, including keeping diagnostic options open and making provisional diagnosis while keeping alternatives in mind (described by use of the exemplar phrase, ‘*I think it’s X, but it might be Y, so I’ll treat you for X while keeping Y in the back of my mind*’) (20). Physicians were observed using these strategies in physician-standardized patient encounters, with greatest use of the strategies in the standardized patient case with the greatest uncertainty (20). Safety-netting was emphasized in a number of papers (18,25,26): this can involve not only explaining any uncertainty surrounding the working diagnosis to a patient, but also giving clear and specific instructions about when patients should return, warning about ‘red flag’ symptoms, explaining the likely timescale of symptoms and also giving advice to third parties (such as carers) if required (18,25).

Communication was not the only technique to manage diagnostic uncertainty identified here: other strategies included ordering investigations, ‘playing for time’ by allowing signs and symptoms to develop, and using external evidence or tools (e.g. risk calculation, guidelines) (2,20,25). How important communication is compared with these other strategies is difficult to quantify: none of the identified studies directly addressed this question, although McKelvie noted that availability and speed of results often limit the practical utility of investigations in primary care (25).

1. There is variation in practice regarding the extent to which diagnostic uncertainty is communicated to patients in primary care.

We found evidence for variation in the extent to which primary care clinicians communicate diagnostic uncertainty to patients.

(a) *Evidence from physicians*: Thompson explored GPs’ opinions on proposed safety-netting guidelines for suspected cancer in UK primary care (18). One of the recommendations was ‘*If the working diagnosis is uncertain, explain the uncertainty to the patient together with the reasons for tests, investigations, watchful waiting, or a trial of management*’ (18). Semi-structured interviews demonstrated that although GPs found this recommendation useful, it is only partially implemented in practice.

Darlow found that in response to patients presenting with low back pain, GPs responded in two ways: by giving a clear diagnosis (e.g. muscle strain) even if they did not necessarily believe this to be the definite diagnosis, or by explaining that the diagnosis was uncertain (24). Zafar also found evidence for variation in the extent to and the manner in which primary care providers discuss diagnostic uncertainty, here in the context of incidental imagining findings (27).

Some studies suggested that primary care physicians do communicate diagnostic uncertainty in certain clinical situations, for example when managing older patients with suspected infection (25), and in the initial management of a patient with vague symptoms suggestive of a rhematological disorder (28).

(b) *Evidence from patients*: Two studies examining patient experiences of late cancer diagnoses revealed a lack of communication about diagnostic uncertainty from primary care clinicians early in the disease presentation (26,29). In Black’s study, patients were often giving a benign working diagnosis from their GP without any discussion of the differential diagnosis (26); in Hauken’s study, many patients reported not being told the reasons for invasive and sometimes painful investigations (29).
(c) *Evidence from observed interactions*: Epstein found that in response to standardized patient prompts about worrying about ‘something serious’, expressions of uncertainty were uncommon: saying ‘*I don’t know’* was the least frequently observed response (21).

Seaburn found that physicians’ responses to ambiguous symptoms in standardized patients were categorized into two primary patterns: high partnering (*n* = 10 physicians) and usual care (*n* = 13 physicians) (22). High partnering physicians were more likely to acknowledge the ambiguous nature of the patients’ symptoms, for example by directly acknowledging diagnostic uncertainty. In contrast, usual care physicians tended not to acknowledge uncertainty, and were more likely to offer a diagnosis earlier in the consultation. These physicians often denied ambiguity, reframing presentations to have an unambiguous meaning.

Robins found evidence for limited communication of diagnostic uncertainty in real consultations: ‘diagnosis rationale’ (sharing personal thinking about patient’s diagnosis, especially what symptoms or signs are suggestive of a condition or distinguish one possible condition from another) was infrequently engaged with as proportion of clinic time (<1.0% mean encounter time) (19).

Overall, the studies identified here suggest that there is considerable variation between different clinicians in whether they communicate diagnostic uncertainty. Several studies suggest that information about diagnostic uncertainty is infrequently shared, or that information is presented in such a way to give a false sense of certainty.

1. A variety of reasons influence the decision to (not) communicate diagnostic uncertainty.

Six studies in our review provided information about *why* primary care physicians decide whether to communicate diagnostic uncertainty (2,16–18,28,30). Time constraints as a barrier to communicating information about diagnostic uncertainty were discussed (18), as was the perception that patients are intolerant of uncertainty (24), preferring ‘*black and white answers*’ (18). The perception that communicating diagnostic uncertainty could have a negative impact on patient confidence was discussed in two studies (2,16). Certain physician characteristics have been shown to influence their disclosure of information about uncertainty: neuroticism was negatively associated with communicating uncertainty, whilst extraversion, agreeableness, conscientiousness and openness to experience were positively associated with it (17).

In two of the studies identified, the importance of communicating about diagnostic uncertainty was discussed in the context of the principles of openness and honesty: physicians emphasized the importance of such communication in managing patient expectations (28) and building trust (28,30).

(5) The evidence regarding the effects of (not) communicating diagnostic uncertainty is mixed.
(a) *Impact of communicating diagnostic uncertainty*: As O’Riordan notes, although shared decision-making encourages the communication of uncertainty with patients, the impact of this on patients ‘*has not been researched to any great degree*’ (2). The evidence we identified on the effects of communicating diagnostic uncertainty was mixed, which may in part be due to inconsistencies in its definition.

Ogden reported that verbal expressions of uncertainty—which, in their study, included admissions of lack of knowledge—have a detrimental effect on patient confidence, which is underestimated by GPs (16). Behavioural expressions of uncertainty, such as looking up information or asking advice from a colleague, were viewed as benign or even beneficial by both doctors and patients.

The idea that communicating diagnostic uncertainty might have a detrimental effect on patient satisfaction or confidence is countered by Epstein’s study which observed physician-standardized patient interactions, and found that physicians’ expressions of uncertainty (‘I don’t know…’), although uncommon, were not associated with lower patient ratings of the physician (21).

In two studies, participants speculated about the importance of honestly discussing uncertain diagnosis with patients in order to maintain patient–physician trust (28,30); neither of these studies directly measured this assertion.

(b) *Impact of* not *communicating diagnostic uncertainty*: Four studies highlighted the negative consequences of *not* communicating information about diagnostic uncertainty to patients (26,28–30). Black found that when primary care clinicians give patients a working ‘benign diagnosis’ without disclosing their uncertainty, patients may be disempowered and/or inappropriately re-evaluate their symptoms, thus delaying re-presentation and therefore diagnosis (26). Two further studies linked failing to adequately communicate the provisional nature of a working diagnosis, and when an ordered treatment is empiric, to diagnostic error/delay (23,28).

In a study of young adult cancer patients, most participants reported that the diagnostic process was characterized by ‘traumatic uncertainty based on a profound lack of information and living in suspense’ (29). For these patients, a lack of communication about the suspected cancer diagnosis was a source of anxiety; one participant commented, ‘*I wish that they had told me what they suspected… and given information… I went home and searched all my symptoms on the Web. And that’s much worse than being told that we suspect this or that… I felt that they REFRAINED from telling me… It’s worse not to know*’ (29).

#### What are the ethical issues associated with communicating (or not communicating) diagnostic uncertainty in this setting?

No primary ethical analyses of the effects of communicating, or not communicating, diagnostic uncertainty in the primary care setting were identified. Yet, the studies discussed above—which examined the motives and consequences of communicating diagnostic uncertainty—all have clear ethical implications, even if they were not explicitly discussed. We therefore performed a secondary ethical analysis of the empirical literature in order to better explore the relevant ethical issues.

(1) Patient autonomy and shared decision-making.

Autonomous decision-making requires individuals to be adequately informed with regard to the decision being made; not communicating clearly about diagnostic uncertainty can be disempowering for patients, and can limit their autonomy (26). Attempts to involve the patient in the decision-making process may involve discussing the degree of diagnostic uncertainty, something best done with good communication skills in the context of a trusting doctor–patient relationship (2). In primary care, clinicians often have longer relationships with their patients compared with secondary care; the ability to consider the patient as an individual and take their background into account could facilitate discussions about diagnostic uncertainty (2). Primary care clinicians who encourage patient participation in decision-making might thus be able to ‘*embrace ambiguity and problem solve together in the context of the patient’s illness and life experience*’ (22).

There is, however, a conflict between respecting patient autonomy—which, in its most absolute terms, would include sharing all uncertainty with the patient to facilitate informed decision-making—and physician autonomy to manage resources at a population level or professional autonomy, to withhold treatments they think will not be beneficial.

1. Beneficence and non-maleficence: does discussing diagnostic uncertainty produce harm or benefit for patients?

The studies identified in this review provided mixed evidence on the impact of communicating diagnostic uncertainty: one study suggested a detrimental effect on patient confidence/satisfaction (16), whilst another found no impact (21). Two papers discussed the potential importance of communicating diagnostic uncertainty in maintain patient trust, but did not provide empirical evidence of this (28,30). The potentially harmful effects of *not* discussing diagnostic uncertainty were highlighted in four studies (26,28–30). As Ogden notes, ‘*current writings in medicine…encourage the expression of uncertainty yet it remains unclear whether this would have a positive or detrimental impact upon the patient*’ (16).

1.  Over-investigation: unintended harm and justice considerations.

Trying to provide (often unrealistic) epistemological or biomedical certainty can cause unintended harm. O’Riordan states: ‘*In order to use tests rationally and reasonably, doctors need to be able to tolerate a certain amount of uncertainty*’ (2). Investigation is a strategy for managing diagnostic uncertainty, yet the ethical implications of inappropriate investigation are important to consider (31). The impact of explicitly discussing diagnostic uncertainty with patients on subsequent diagnostic test use in primary care has not been explored.

## Discussion

### Summary

This review reveals that although the importance of developing strategies to manage diagnostic uncertainty in primary care—including communication—has been noted, diagnostic uncertainty is not always discussed with patients in practice. The reasons behind decisions about (not) communicating diagnostic uncertainty are mixed, as is evidence on the effects of the (non) communication on patients; there is a paucity of work examining patient preferences for this communication. The empirical literature is limited by inconsistencies in how diagnostic uncertainty is defined and measured. The ethical implications associated with the (non) communication of diagnostic uncertainty—respecting the autonomy of patients, acting in patients’ best interests and avoiding harm, over-investigation—were evident from secondary analysis of the identified studies.

### Further discussion

(1) Methodological limitations.

The heterogeneity in measurement and definition of diagnostic uncertainty has been noted by others. Bhise discussed the lack of a widely accepted definition, proposing the following: ‘*subjective perception of an inability to provide an accurate explanation of the patient’s health problem*’ (32). They also noted that ‘*although different methods have been used to study diagnostic uncertainty in clinical practice, evidence is limited on which of these is the most useful*’ (32). Tools for measuring the communication of diagnostic uncertainty are in general under-developed and not widely validated, something which significantly limits the current empirical evidence base (32–34).

1. A need for more patient-focussed empirical research.

One study in this review suggests that communicating diagnostic uncertainty can have a negative impact on patient satisfaction (16), whilst another suggested no impact (21); the literature in acute secondary care settings is similarly mixed (35–38). There are a number of factors which could explain the inconsistencies: methodological limitations (as discussed above); the relatively small body of existing literature on this complex topic; and the fact that in different clinical situations communicating diagnostic uncertainty might have different effects on patients. Very little work has directly examined patient preferences for the communication of diagnostic uncertainty in primary care; studies in other settings suggest a desire for this information, and a mismatch between doctor *perceptions* of patient informational desires and patient reported informational desires (39–41). A recent review concluded that a particular challenge is the ‘*communication of uncertainty in a meaningful way that enhances trust in the patient-provider relationship, and improves decision-making and healthcare outcomes*’ (34); more evidence on what information patients want could help achieve this.

1.  The importance of acknowledging and learning to deal with uncertainty.

Many of the papers here acknowledged that diagnostic uncertainty is routine in primary care (2,17,18,20,22). That primary care clinicians must develop skills to manage it has been noted elsewhere, particularly in terms of reducing stress and preventing over-investigation (3,42,43). Teaching in medical school engrains the idea that there is a single correct answer, and that certainty is always obtainable—this is not the reality in practice, and several papers have called for changes to be made to medical training to reflect this (2,3,44). Empirical studies support the idea that medical training does not adequately cover the communication of uncertainty to patients (36,38).

In primary care, there is a reasonable body of evidence linking intolerance to uncertainty to anxiety, stress and burn-out (1,45,46). The relationship between communicating diagnostic uncertainty and physician’s affective responses to uncertainty has not been examined in detail. Although intolerance to uncertainty is associated with a reluctance to disclose uncertainty (45), it is not clear what impact explicitly sharing diagnostic uncertainty with patients might have on physicians’ own discomfort. In one study of emergency residents, some doctors described having a positive conceptualization of diagnostic uncertainty, discussing it with patients as a safeguard against diagnostic error: ‘*I think that’s important that they understand that medicine is not an exact science*’ (47). Promoting open communication with patients as a way of positively managing diagnostic uncertainty could be useful in equipping primary care clinicians to better manage uncertainty in practice—more research is needed to explore this.

1.  Ethical analyses are under-developed.

The specific ethical issues surrounding the communication of diagnostic uncertainty (as opposed to other types of uncertainty) have not been examined in detail; we did not identify any primary ethical analyses in our review. In particular, there is a need for further empirical work to help guide the development of ethical guidelines which may help to support doctors in their communication of diagnostic uncertainty in practice. A better understanding, for example, of whether communicating this information has a beneficial or harmful effect on patients, would facilitate a more nuanced discussion of the ethical issues highlighted here.

### Strengths and limitations

This was a comprehensive review of the literature on the ethical issues surrounding communicating diagnostic uncertainty in primary care. However, the nature of the studies identified were heterogeneous and allowed only descriptive analysis. All of the studies were conducted in western cultures; by only including published papers in English, we may have missed research more relevant to other cultural settings.

## Conclusion

Clinicians working in primary care experience diagnostic uncertainty routinely. This review has highlighted significant gaps in the existing literature: there is a need for explicit ethical and patient-centred empirical analyses on the effects of communicating diagnostic uncertainty on patient experience and diagnostic testing. Consensus on how diagnostic uncertainty should be defined, and greater research into tools for its measurement, would help to strengthen the existing empirical evidence base. More work is needed in particular to gain insights into patients’ perspectives of diagnostic uncertainty; this will help identify which communication and investigation approaches are desired and beneficial to patients.

## Supplementary Material

cmab023_suppl_supplementarry_Material

## Declaration

Funding: ZF is funded by the Wellcome Trust (grant number 208213/Z/17/Z). CLC is funded by an NIHR Academic Clinical Fellowship. ZF and CLC are based in The Healthcare Improvement Studies Institute (THIS Institute), University of Cambridge. THIS Institute is supported by the Health Foundation, an independent charity committed to bringing about better health and healthcare for people in the UK.

Ethical approval: not available.
